# The Effect of Dynamic, In Vivo-like Oxaliplatin on HCT116 Spheroids in a Cancer-on-Chip Model Is Representative of the Response in Xenografts

**DOI:** 10.3390/mi13050739

**Published:** 2022-05-06

**Authors:** Job Komen, Sanne M. van Neerven, Elsbeth G. B. M. Bossink, Nina E. de Groot, Lisanne E. Nijman, Albert van den Berg, Louis Vermeulen, Andries D. van der Meer

**Affiliations:** 1 BIOS Lab on a Chip Group, MESA+ Institute for Nanotechnology, University of Twente, 7500 AE Enschede, The Netherlands; a.vandenberg@utwente.nl; 2 Laboratory for Experimental Oncology and Radiobiology, Center for Experimental and Molecular Medicine, Cancer Center Amsterdam and Amsterdam Gastroenterology, Endocrinology and Metabolism, Amsterdam University Medical Centers, 1105 AZ Amsterdam, The Netherlands; s.m.vanneerven@amsterdamumc.nl (S.M.v.N.); e.g.b.m.bossink@utwente.nl (E.G.B.M.B.); n.e.degroot@amsterdamumc.nl (N.E.d.G.); l.e.nijman@amsterdamumc.nl (L.E.N.); l.vermeulen@amsterdamumc.nl (L.V.); 3 Applied Stem Cell Technologies, TechMed Centre, University of Twente, 7500 AE Enschede, The Netherlands; andries.vandermeer@utwente.nl

**Keywords:** cancer-on-chip, xenograft, microfluidic, colorectal cancer, pharmacodynamics, pharmacokinetics, drug efficacy, oxaliplatin

## Abstract

The cancer xenograft model in which human cancer cells are implanted in a mouse is one of the most used preclinical models to test the efficacy of novel cancer drugs. However, the model is imperfect; animal models are ethically burdened, and the imperfect efficacy predictions contribute to high clinical attrition of novel drugs. If microfluidic cancer-on-chip models could recapitulate key elements of the xenograft model, then these models could substitute the xenograft model and subsequently surpass the xenograft model by reducing variation, increasing sensitivity and scale, and adding human factors. Here, we exposed HCT116 colorectal cancer spheroids to dynamic, in vivo-like, concentrations of oxaliplatin, including a 5 day drug-free period, on-chip. Growth inhibition on-chip was comparable to existing xenograft studies. Furthermore, immunohistochemistry showed a similar response in proliferation and apoptosis markers. While small volume changes in xenografts are hard to detect, in the chip-system, we could observe a temporary growth delay. Lastly, histopathology and a pharmacodynamic model showed that the cancer spheroid-on-chip was representative of the proliferating outer part of a HCT116 xenograft, thereby capturing the major driver of the drug response of the xenograft. Hence, the cancer-on-chip model recapitulated the response of HCT116 xenografts to oxaliplatin and provided additional drug efficacy information.

## 1. Introduction

Cancer is the leading cause of death in the Western world [1]. Continuous improvements in treatment have extended survival times; nevertheless, high attrition rates in clinical trials of novel drugs hamper treatment improvement [2,3]. One reason for high attrition rates is the limited translational quality of in vitro and animal models [4].

One of the most used animal models in cancer drug discovery is the mouse xenograft model [5]. In this model, human cancer cells are implanted in an immune-deficient mouse. The xenograft adds certain physiologic aspects compared to in vitro assays, such as pharmacokinetics, homeostasis, and three-dimensional, vascularized tumor growth. However the predictive power of the model toward human cancer response has limitations due to the reduction in biological complexity, species differences, and experimental variation [6]. Despite these shortcomings, the xenograft model remains a key assay for in vivo efficacy testing of novel drugs before clinical trials.

Basic human cell culture models are no direct alternative for xenograft models in preclinical efficacy testing, as they are incapable of capturing all relevant aspects of an in vivo tumor. Recently, microfluidic ‘cancer-on-chip’ technology has led to major improvements in modeling tumor physiology in vitro. Cancer-on-chip models can recapitulate essential in vivo characteristics of cancer, such as continuous perfusion to maintain homeostasis, angiogenesis, controllable oxygen and nutrient gradients, interaction with stromal cells, and mechanical stimulation [7,8,9,10,11,12]. Recently, the possibility to offer dynamic, in vivo-like drug concentrations has been added to this repertoire [12,13,14]. Despite all these promising aspects, cancer-on-chip technology has not yet been sufficiently validated to significantly replace existing preclinical, in vivo assays. Validation against xenograft models will be an essential step for cancer-on-chip models to be used as valid alternatives in preclinical efficacy testing. Moreover, such validation will offer an indispensable foundation for further development of even more advanced cancer-on-chip models with added human complexity [15]. 

Comparison of the drug response in cancer-on-chip models to xenografts is budding, and multiple studies have performed such side-by-side experiments [16,17,18]. These first studies were based on cancer-on-chip models that included vascularization, stromal cell coculture [18,19], patient-derived cells [16], and dynamic drug concentrations [17]. The results of the side-by-side comparisons were highly encouraging, with cancer-on-chip models and xenografts based on the same cells responding similarly upon treatment with the same drugs. However, these early studies focused on live–dead stains [16], volume-based growth readouts [17], in situ immunofluorescence [17], or gene expression [18] for comparison between cancer-on-chip and xenograft models. An important readout in xenograft studies is provided by histopathology and immunohistochemistry, which provides data on cancer cell morphology and tissue structure. Furthermore, immunostaining of tissue sections reduces the probability of signal distortion compared to in situ immunofluorescence, where dampening of the signal can occur due to the thickness of the cancer tissues [20]. Therefore, side-by-side histological analysis of both cancer-on-chip and xenograft models will be an indispensable step in the validation of cancer-on-chip models.

Here, we performed a side-by-side comparison between the response to oxaliplatin of HCT116 colorectal cancer spheroids in a cancer-on-chip model versus HCT116 xenografts in existing studies. We evaluated the effect of oxaliplatin on volume growth and proliferation and apoptosis markers. Moreover, we explored how histological information can be used to scale the overall growth data of spheroids in the cancer-on-chip model to xenografts to provide an improved comparison with data from the in vivo xenograft.

## 2. Materials and Methods

### 2.1. Device Design and Fabrication

**Figure 1:**
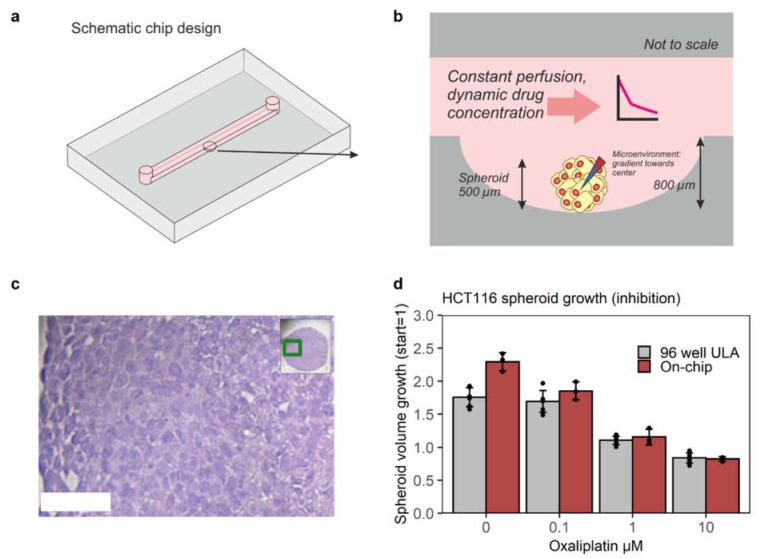
**Design and validation of the chip system for mimicking oxaliplatin efficacy in a HCT116 xenograft model.** (**a**,**b**) Chip design. The two-part chip consists of a 21 × 2 × 1 mm (l × w × h) top channel for continuous perfusion and dynamic drug concentrations and a U-shaped well for spheroid placement and growth. (**c**) H&E staining shows low differentiation and little extracellular matrix in the spheroid on-chip, as in HCT-116 xenografts. Scale bar = 50 µm. (**d**) Growth inhibition after 48 h exposure to constant concentrations of oxaliplatin is not confounded by the chip, although control growth is slightly higher, as shown by comparison to a 96-well ultralow-attachment (ULA) plate; *n* = 3 spheroids for on-chip, *n* = 6 spheroids for 96 well; error bars represent the standard deviation (SD).

The microfluidic chip consisted of two detachable parts (Figure 1a,b). The top part contained a 21 × 2 × 1 mm (length × width × height) straight channel for continuous perfusion of nutrients and drug. The bottom part contained a single U-shaped well for holding a single spheroid. The well had a diameter of 2 mm and a depth of 0.8 mm to facilitate placement and growth of spheroids from 0.5 mm to 1 mm diameter (Figure 1a,b).

Chip parts were made of polydimethylsiloxane (PDMS). Chips were fabricated using PDMS in a 10:1 weight ratio of base to curing agent (Sylgard 184, Dow Corning, USA). The PDMS was poured on a micro-milled mold (Datron Neo, Germany) and cured at 60 °C overnight. Inlet and outlet holes were punched using Harris UniCore punchers of 1 mm diameter. Medical-grade adhesive, double-sided tape AR Care 8939 (Adhesive Research, Limerick, Ireland), 0.11 mm thickness, was applied to the bottom part before coating the well. For ensuring non-adhesion of the spheroids to the well, a coating of Pluronic F127 was used [21]. Pluronics attach to the hydrophobic PDMS with a central hydrophobic block, and the hydrophilic tails form a hydrophilic brush which prevents binding of proteins and cells [21,22]. Pluronics have been shown to prevent cell adhesion for up to 4 weeks, and they remain intact in microfluidic channels after flow [23,24]. Pluronic F127 has been used to prevent adhesion of spheroids in chips for up to 10 days [21]. Pluronic F127 20 mg/mL in deionized (DI) water was applied to the wells and incubated for 2 h at 37 °C [21]. To ensure homogeneous coating, Pluronic F127 was prevented from drying out by a droplet volume of 6 µL and the placement of a PDMS cap over the well. Chips were placed in a box inside the incubator with Kimwipes soaked in phosphate-buffered saline (PBS, ThermoFisher, Waltham, MA, USA) to prevent drying out. After incubation, the Pluronic was removed, and the well was flushed with 2 µL of medium before placing the spheroids.

### 2.2. Cell and Spheroid Culture

HCT-116 colorectal cancer cells (Sigma-Aldrich, Burlington, MA, USA), with a passage number under 30, were cultured in McCoy 5A medium (ThermoFisher, USA) with 5% fetal bovine serum (FBS, ThermoFisher) and 1% penicillin–streptomycin (ThermoFisher, USA). Four days before the start of the chip experiment, 2000 HCT116 cells in 200 µL of medium per well were placed in a Nunclon Sphera ultralow-absorption round-bottom plate (ThermoFisher, USA). Cells were spun at 390 G for 5 min and placed in a humidified incubator at 37 °C.

### 2.3. Chip Experiments

Spheroids were collected from the 96 well plate with a wide-bore P200 pipette tip set at 50 µL (VWR, Radnor, PA, USA). In several seconds, the spheroid sunk to the bottom of the tip, and the tip was subsequently brought in contact with the medium in the well, resulting in the transfer of the spheroid. Subsequently, the top part of the chip was placed on the adhesive tape, which was already attached to the bottom part. Chips were filled with medium at a flow of 20 µL/min. Chips were continuously perfused at a flow rate of 2 µL/min using a syringe pump (Harvard PhD2000, Fargo, ND, USA).

### 2.4. Histopathology

Spheroids were taken off-chip for histopathology by removing the top part of the chip, by inserting tweezers between the top part and the adhesive tape, without touching the channel with the tweezers. A PDMS ring with an inner diameter of 8 mm and a thickness of 3 mm was placed around the well and gently filled with 150 µL of PBS. The spheroid was harvested with the p200 wide bore pipette tip and transferred to 4% paraformaldehyde (PFA, Sigma, USA) in PBS. Spheroids were placed in 70% alcohol overnight, dehydrated, transferred to a biopsy foam pad, embedded in paraplast, and cut into 0.4 µm sections. Tissue sections were rehydrated and stained with hematoxylin and eosin (H&E) to evaluate the tissue structure or a diaminobenzidine (DAB) staining for apoptosis (cleaved Caspase-3) or proliferation (Ki67) with a hematoxylin counterstain. Antibodies used for the DAB staining were primary antibodies cleaved Caspase-3 (ASP-175) (1:1000, Cell Signaling #9661, USA) or Ki67 (1:1000, Sigma #SAB5500134, USA) and secondary anti-rabbit antibody linked to horseradish peroxidase (HRP). Slides were dehydrated and mounted with Pertex (VWR, Amsterdam, The Netherlands). 

### 2.5. Imaging and Analyses

Brightfield images of the spheroids on-chip were taken at t = 0, t = 2 days, and t = 7 days at 10× magnification (Leica DM IRM, Wetzlar, Germany). Spheroid size was determined with open-source software Fiji/ImageJ using the algorithm developed by Ivanov et al. (Supplementary Figure S1) [25]. Spheroid growth inhibition was based on the formula as described in the xenograft literature [26], and was defined as

$$1-\frac{\mathrm{average}(Volume\;end_{treatment}-Volume\;start_{treatment})}{\mathrm{average}(Volume\;end_{control}-Volume\;start_{control})}.$$

Spheroid cell counts were carried out by harvesting the spheroids from the chips as described in the histopathology section; however, instead of fixation, spheroids were placed in a 96-well plate in 100 µL of trypsin for 20 min at 37 °C. After resuspension, cells were counted with a Luna automated cell counter (Logos Biosystems, Anyang-si, South Korea).

Immunohistochemistry analyses were conducted with open-source software Qupath on 20× brightfield images [27]. For Ki67 detection, colors were first deconvoluted for each image with the “estimate stain vectors” function. Nucleus detection was based on the parameters used by Robertson et al. [28]. The cutoff for Ki67 positivity was 0.3. CC3 positivity was based on the percentage of positive pixels within the spheroid at a cutoff value of 0.5.

### 2.6. Xenograft Data

To compare growth inhibition on-chip to published xenograft studies, a literature search was conducted. Inclusion criteria were as follows: indexation in PubMed, analysis period, and treatment start at a tumor starting volume of 50 mm^3^ or larger, subcutaneous placement of tumors, and more than one treatment cycle during the analysis period of 2–5 weeks [26,29,30,31,32,33,34]. An overview of tumor growth (inhibition) data is provided in Supplementary Table S1 and Supplementary Figure S2.

To compare the histology and immunohistochemistry between the untreated on-chip spheroids and HCT116 xenografts, xenograft specimens obtained in an existing xenograft experiment were used [35]. Briefly, 1 × 10^6^ human CRC cells were injected subcutaneously in NOD/SCID IL2R gamma^−/−^ (NSG) mice. When tumors reached a volume of ~300 mm^3^, mice were sacrificed and xenografts were stored in formalin-fixed, paraffin-embedded (FFPE) blocks.

## 3. Results and Discussion

### 3.1. Chip Design and Validation for Mimicking Xenograft Drug Response On-Chip

**Figure 2:**
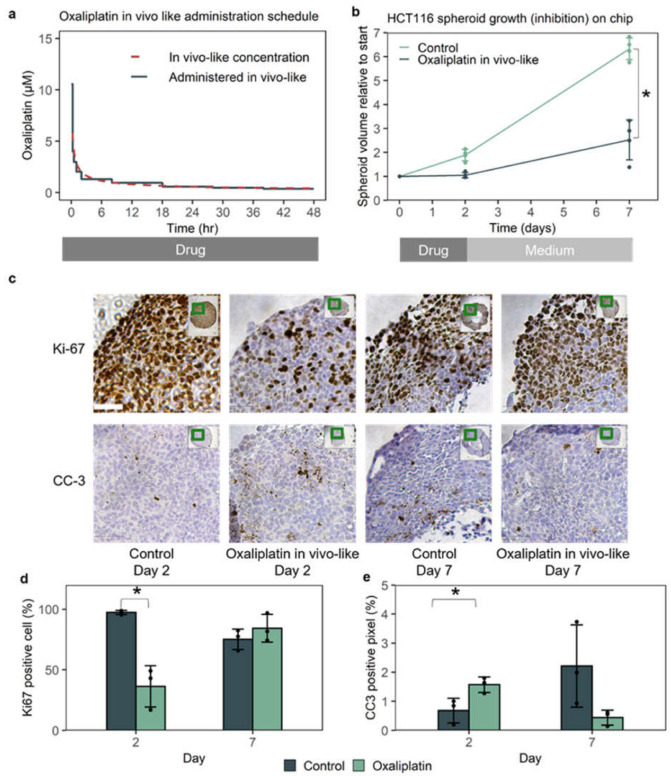
**In vivo-like oxaliplatin leads to 70% growth inhibition on-chip based on spheroid volume, with a temporary reduction of proliferation and increase in apoptosis.** (**a**) In vivo-like oxaliplatin concentration over time found in mice [36], corrected for protein binding [37], translated into an in vivo-like drug administration schedule. (**b**) Dynamic, in vivo-like oxaliplatin concentration led to temporary, partial growth inhibition of the spheroid. *N* = 4 spheroids per condition. Error bars represent the SD. * *p* < 0.001 according to a two-sided, unequal variance *t*-test. (**c**) In vivo-like oxaliplatin reversibly decreased proliferation (Ki67) for 2 days, and increased apoptosis (CC-3), albeit from a low level. Scale bar = 50 µm. (**d**,**e**) Quantification of staining with Qupath [27,28] between control and treated chips after 2 and 7 days. Depicted data points represent sections from two separate spheroids. * *p* < 0.05 according to two-sided, unequal variance *t*-test with *N* = 3 sections per condition. Error bars represent the SD.

To mimic drug efficacy in a xenograft model on-chip, important biological factors and readouts should be comparable [15]. Injection of the colorectal cancer cell line HCT116 below the skin of the mouse led to a fast-growing tumor with poor differentiation (Supplementary Figure S3). When the tumor achieved a measurable volume (50–300 mm^3^), oxaliplatin was administered, typically one to several times a week for multiple cycles [26,29,30,31,32,33,34]. Oxaliplatin concentration in the mouse was characterized by a peak and an exponential decline, with limited free, non-protein-bound drug, measurable 48 h after administration (Figure 2a) [36,37]. During the experiment, the diameter of the tumor was measured with calipers, and the volume was derived. Growth inhibition was based on the volume at the end of the experiment of the control group versus the treated group. Isolated tumors were further evaluated with histopathology, e.g., on proliferation and apoptosis markers [26,29,30,31,32,33,34].

To incorporate these key biological parameters and readouts, especially histopathology, a detachable two-part chip was designed (Figure 1a,b). The top part consisted of a straight channel to supply continuous perfusion of nutrients and dynamic drug concentrations [14]. The bottom part contained a 2 mm diameter, 0.8 mm deep U-shaped well to facilitate placement and unimpeded growth of spheroids (Figure 1b). The well was able to hold the spheroids under flow without the need for fixation with the extracellular matrix. 

The chip was reversibly bonded by medical-grade adhesive tape to enable off-chip standard histopathology. Spheroids were taken out of the chip, formalin-fixed, paraffin-embedded (FFPE), cut in sections, and stained with hematoxylin–eosin (HE), or hematoxylin and markers for proliferation (Ki67) and apoptosis (cleaved Caspase-3, CC3). HE staining showed that spheroids contained poorly differentiated cancer cells, with limited extracellular matrix, as observed in viable regions of the HCT116 xenografts (Figure 1c, Supplementary Figure S3). 

To mimic oxaliplatin concentrations over time found in the blood of mice, dynamic control of solute concentrations was needed. Dynamic control of oxaliplatin with a molecular weight of 397 Dalton (Da) and estimated diffusion coefficient of 8.2 × 10^−6^ cm^2^·s^−1^ was validated with fluorescein, a small molecule with a molecular weight of 332 Da and diffusion coefficient estimates of 4–5.7 × 10^−6^ cm^2^·s^−1^ [38,39]. Concentration steps similar to oxaliplatin were programmed, and the fluorescence signal was quantified and compared to expected fluorescence changes. Measured fluorescence follows expected fluorescence, taking into account expected lags in concentration changes due to Poiseuille flow (Supplementary Figure S6). Dynamic concentration control of oxaliplatin on-chip has also been described elsewhere in more detail [14].

The well was coated with Pluronic F127 to prevent adhesion and non-spheroidal growth of the spheroid [21]. The U shape of the well allowed for central placement of the spheroid-by-spheroid transfer via fluid–fluid contact. Central placement prevented automated imaging challenges and distortion of diffusion of nutrients to the spheroid. 

An algorithm in Fiji/ImageJ based on earlier work was used to quantify the spheroid surface and derive volume (growth) (Supplementary Figure S1) [25]. Due to the non-adhesion of spheroids, the outer diameter accurately reflected spheroid volume. Trypsinization of spheroids of different sizes and subsequent automated cell counting confirmed the correlation between volume derived from the outer diameter and cell number growth (Supplementary Figure S4). 

The chip system should not confound control growth and drug effect [40]. Ideally, cancer-on-chip technology can bridge the gap between standard in vitro assays and in vivo models. It is especially important to verify that cancer cell growth is not disrupted on-chip, and that the chip does not interfere with drug efficacy. Therefore, spheroids were exposed to constant oxaliplatin concentrations on-chip and in an ultralow-attachment plate with U-shaped wells (‘Sphera’). Average starting diameters across treatment conditions on-chip and in the well plate ranged from 0.45 to 0.48 mm (Supplementary Figure S5). Although control growth was slightly higher on-chip (2.3 fold in 48 h) than growth in Sphera plates (1.8 fold), likely due to constant perfusion, growth under treatment was very similar (Figure 1d). A two-way analysis of variance (ANOVA) for unbalanced data [41] led to *p*-values of 2.2 × 10^−16^, 1.5 × 10^−4^, and 3.2 × 10^−4^ for the factors of oxaliplatin dose, chip versus 96-well plate, and interaction, respectively. The significance of interaction is driven by the control growth conditions, which also drives the overall significance of the difference between chip and well plates, as the *p*-values of chip versus 96-well plate and interaction were 0.15 and 0.29, respectively, when the control condition was left out of the ANOVA. Hence, the chip system did not negatively affect cancer spheroid growth or efficacy of oxaliplatin. 

The growth (inhibition) results for validating the chip system also clearly showed a difference in growth of HCT116 cells in spheroids versus monolayers (2D). Untreated doubling time increases from 20 h to 48 h for HCT116 spheroids with a typical diameter of 0.5 mm [42].

### 3.2. In Vivo-like Oxaliplatin Led to 70% Growth Inhibition On-Chip, with a Temporary Halt of Growth

To mimic growth inhibition in xenograft studies, drug exposure in mice needed to be recapitulated. Dosages in in vivo studies varied from 5–20 mg/kg per week, given in 1–3 dosages per week [26,29,30,31,32,33,34]. Here, we used oxaliplatin concentrations measured in blood plasma in mice over time after a single dose of 8 mg/kg [36]. As the only drug which is not bound to plasma proteins is active [43], the total oxaliplatin concentration was corrected for the fraction of oxaliplatin bound to plasma proteins. As the free oxaliplatin concentration over time in mice was found to be only 33% of total drug concentration, total plasma levels of oxaliplatin found in mice were decreased by 67%, which resulted in the unbound oxaliplatin concentration over time [36,37]. The in vivo (unbound) oxaliplatin concentration over time was translated to a drug administration schedule, and both are shown in Figure 2a. 

Growth inhibition is the primary readout of xenograft experiments. Exposure of HCT116 spheroids to in vivo-like oxaliplatin on-chip resulted in growth inhibition of 70%, as measured over a 7 day period (Figure 2b). Growth inhibition of 70% found on-chip matches well with average growth inhibition of 60% (range 30–80%) found in xenografts (Supplementary Figure S2, Table S1) [26,29,30,31,32,33,34].

Immunohistochemistry can give further insight into the molecular mechanisms of growth inhibition of the drug [32,44]. Immunohistochemical markers were quantified for the entire spheroid section using Qupath software, which has been used for scoring Ki67 in clinical breast cancer samples (Figure 2c,d, Supplementary Figures S7 and S8) [27,28]. Immunohistochemistry indicated that drug treatment led to both a reduction in proliferation marker Ki67 and an increase in apoptosis marker CC3 after 2 days compared to control spheroids at 2 days (Figure 2c–e). Oxaliplatin treatment of HCT116 xenografts also led to a decrease in Ki67 and increase in CC3 [32,33,44]. 

The in vivo-like drug exposure led to a growth stop for the first 2 days, after which growth recovered. The recovery of growth was accompanied by an increase in the proliferation marker Ki67 and a decrease in CC3 after 7 days, as compared to treated spheroids after 2 days (Figure 2c–e). Comparing the Ki67 and CC3 status of the treated spheroid with the untreated spheroid after 7 days was less straightforward, as the untreated spheroid had, due to its size, a decreased proliferation rate and a higher baseline apoptosis, especially surrounding the necrotic core.

To our knowledge, the temporary growth reduction and subsequent growth recovery have not been described for HCT116 xenografts treated with oxaliplatin in in vivo studies, possibly due to the limited precision of measuring tumor volume through the skin with calipers, on a millimeter scale, and the number of animals needed for histological analyses at multiple timepoints. For HCT116 xenografts treated with gemcitabine, daily histological sections did show a similar full growth stop and gradual recovery over six days, as measured with BrdUrd [45]. Knowledge of recovery times of cancer cells in vivo could support future design of clinical dosage schedules, which, for example, is once per 2–3 weeks for oxaliplatin, although side-effects also have to be taken into account [46,47].

### 3.3. The Cancer-On-Chip Model Recapitulates Drug Response as It Is Representative of Proliferating Cells in the HCT116 Xenograft

In the previous section, it was shown that growth inhibition on-chip and in xenografts was comparable. Nevertheless, even though spheroids share similarities with xenografts, such as spheroidal shape and solute gradients, spheroids also differ from xenografts in several ways, e.g., spheroids are avascular and typically have a diameter 10× smaller than xenografts. Furthermore, although large spheroids grow more slowly than 2D cultured cells, spheroids still typically grow faster than xenografts of the same cell line. 

The structural and growth differences between xenografts and spheroids raises the question of whether the spheroid is representative of the whole xenograft tumor or only for specific areas. 

**Figure 3:**
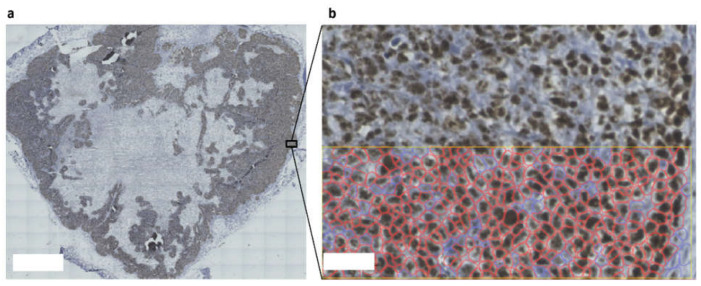
**An HCT116 xenograft with a proliferating shell and a nonproliferating core.** (**a**) HCT116 xenografts stained for proliferation marker Ki67 (brown) contained peripheral regions with high proliferation and large areas of nonproliferating tissue toward the core. Scale bar = 2 mm. (**b**) Proliferating areas were ~80% Ki67-positive. Ki67 quantification was performed by Qupath software; positive cells are shown in red [27]. Scale bar = 50 µm.

The untreated spheroids on-chip were 95% Ki67-positive after 2 days, which declined to ~75% after 7 days (Figure 2c,d, Supplementary Figure S7). Histopathological analysis of untreated HCT116 xenografts showed that only a fraction (46%) of the xenograft was proliferating (Figure 3a, Supplementary Figure S10). The proliferating cells clustered in distinct regions toward the periphery of the tumor, likely due to differences in perfusion and interactions with the host stroma [48,49,50]. These proliferating regions contained ~80% Ki67-positive cells (Figure 3b), whereas the nonproliferating regions contained <5% Ki67-positive cells (Supplementary Figure S12). With H&E staining, the non-proliferative regions were also characterized by eosinophilic staining, nuclear condensation, and loss of nuclei, suggesting necrosis (Supplementary Figure S3) [51].

The presence of proliferating and nonproliferating regions in the HCT116 xenograft not only provides an explanation for the different control growth rates, but can also explain the comparable growth inhibition found in both the cancer-on-chip model and the xenografts. The effect of oxaliplatin on the nonviable, nonproliferating, regions will be limited, as they are not growing, poorly perfused, and already in a (pre)necrotic state (Supplementary Figure S3) [49]. In the viable, proliferating, regions ~80% of cells were Ki67-positive, for which the spheroid-on-chip should be representative. Hence, the effect of oxaliplatin in xenografts is likely dominated by the effect on proliferating cells, which are present on-chip.

### 3.4. A Pharmacodynamic Model Further Validates the Representativeness of On-Chip Growth Inhibition

The representativeness of the spheroid for the proliferating cells in the xenograft provided a qualitative argument for why the drug effect is recapitulated on-chip. A pharmacodynamic (‘the effect of drug on tissue’) model, in which on-chip growth is mapped to the representative parts of the xenograft to predict treated and untreated growth, could further validate the ability of the cancer-on-chip model to mimic the effect of oxaliplatin on HCT116 xenografts, as well as identify translational discrepancies. 

**Figure 4:**
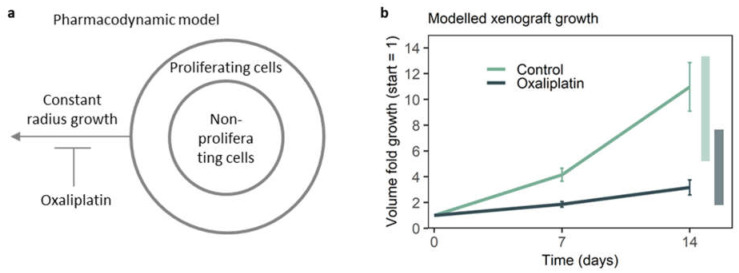
**The pharmacodynamic model of HCT116 xenograft growth.** (**a**) The modeled xenograft consisted of a shell of proliferating cells. Constant radius growth was derived from on-chip spheroid growth and the thickness of the proliferating shell in untreated HCT xenografts (see text). (**b**) Modeled growth with and without treatment. Error bars represents the standard error of the mean (SEM), based on individual growth found in *n* = 4 chips per condition. Shaded bars on the right represent the range found in xenograft studies [26,29,30,31,32,33,34].

As HCT116 xenografts consist of proliferating and non-proliferating cells, the pharmacodynamic model employed here consisted of two populations, a proliferating and nonproliferating subpopulation [52,53]. The subpopulations were geometrically divided (Figure 4a), as the vast majority of proliferating cells were located on the periphery of the xenografts (Figure 3a and Figure 4a, Supplementary Figure S10). Constant radius growth was assumed for the modeled xenograft, as constant radius growth has been described for other colorectal cancer xenografts [50,54] and fits with growth curves for HCT116 xenografts [26,29,30,31,32,33,34].

The formula describing xenograft growth employed here was as follows [50]:

$$V\left(t\right)=4/3\;\pi \;{\left(\alpha t+\beta \right)}^{3},$$

where V is the volume of the tumor in mm^3^ at day t, α represents the radius growth per day in mm/day, and β is the starting radius in mm. Starting volume was set at 100 mm^3^ (β = 2.9 mm), an often-used starting volume in xenograft studies (Supplementary Table S1).

Radius growth per day (*α*) was based on the thickness of the proliferating shell found in xenografts and the on-chip growth of spheroids in the first 2 days (Supplementary Figures S10 and S11 and accompanying data). For untreated modeled xenografts, this resulted in a radius growth of 0.25 mm/day and a tumor volume growth in 14 days from 100 mm^3^ to 1100 mm^3^, while, for modeled xenografts treated with oxaliplatin, the resulting radius growth was 0.11 mm/day and the final tumor volume was 376 mm^3^ (Figure 4b).

Modeled xenograft growth with and without oxaliplatin treatment fell within the range of growth found in xenograft studies (Figure 4b); untreated tumor volume growth in existing HCT116 xenograft studies over 14 days was 5–13-fold, and xenografts treated with oxaliplatin grew 1.5–7.5-fold over 14 days (Figure 4b, Supplementary Table S1) [26,29,30,31,32,33,34]. Hence, the outcome of the pharmacodynamic model for untreated and treated xenograft growth was on the high side, but within the range found in existing xenograft studies. 

Potential reasons for the relatively high growth predictions could be a slightly higher proliferation rate in spheroids in the first 2–3 days, a decline in radius growth per day at higher tumor volumes in xenografts, higher external pressure in xenografts [55] leading to potential compression of the necrotic core during growth, and measurement errors due to interference of skin and connective tissue with caliper measurement [56], which could have a larger influence on smaller starting tumors. 

However, according to the immunohistochemistry showing that on-chip spheroids were representative of the proliferating cells, and the pharmacodynamic model resulting in modeled xenograft growth (inhibition) on the high side but within the range of actual xenograft studies, remaining potential translational discrepancies provide room for future optimization but do not seem to interfere with the ability of the cancer-on-chip model to represent the effect of oxaliplatin on HCT116 xenografts.

With further validation, cancer-on-chip models could, thus, complement cancer xenograft models. Different cell lines, dosage schedules, and drug combinations can be tested for further validation of novel drug efficacy without increasing animal use. Furthermore cancer-on-chip models have the potential to decrease the variability of in-vivo assays as there is less unwanted biological variation from the animal host. Moreover, continuous monitoring of cancer growth-on-chip could elucidate drug exposure and response relations. To mimic human cancer more closely, in addition to expanding cell lines to have greater coverage of clinical variety, human aspects can be added in a stepwise fashion, such as human pharmacokinetics, stroma, and immune components, as such cancer-on-chip models have the potential to progressively complement and substitute the cancer xenograft model.

## 4. Conclusions

A cancer-on-chip model was designed and validated to incorporate key elements of the cancer xenograft assay. On-chip growth inhibition of HCT116 colorectal cancer spheroids at in vivo-like oxaliplatin concentrations was comparable to in vivo xenograft growth inhibition, while providing novel insights into the temporal response. Furthermore, immunohistochemistry performed off-chip indicated a decrease in proliferation marker Ki67 and an increase in apoptosis marker CC3 right after treatment. 

As the size and structure of xenografts and spheroids differ, we further evaluated the representativeness of the spheroid on-chip. Immunohistochemical staining suggested that the on-chip HCT116 spheroid was representative of the proliferating cells in the outer shell of the xenograft, which likely determine the growth and growth inhibition in the xenograft. Furthermore, a pharmacodynamic model with a proliferating and nonproliferating cell population, in which constant radius growth was based on growth (inhibition) found on-chip, resulted in comparable growth (inhibition) to HCT116 xenografts treated with oxaliplatin, thus providing additional support for the translational relevance of the cancer-on-chip model. 

## Data Availability

Not applicable.

## Supplementary Materials

The following supporting information can be downloaded at https://www.mdpi.com/article/10.3390/mi13050739/s1. Figure S1: Spheroid size determination by the Fiji algorithm. Table S1: Xenograft studies used for comparing growth inhibition and growth. Figure S2: Growth inhibition from existing HCT116 xenograft studies plotted against dose per week. Figure S3: HCT116 xenograft tissue stained with hematoxylin-eosin (HE). Figure S4: Correlation between image based tumor volume derivation of the spheroids and automated cell count after trypsinization. Figure S5: Equality of spheroid start diameter per treatment condition, on-chip and in the 96 well plate (“sphera”). Figure S6: Validation of dynamic drug control on chip. Figure S7: Ki67 (proliferation) quantification of HCT116 spheroid sections in Qupath. Figure S8: Detection of apoptosis marker cleaved caspase 3 (CC3). Figure S9: Limited cleaved caspase 3 (CC3) staining at the tumor edge of untreated xenografts. Figure S10: Macroscopic view of HCT116 xenografts stained with HE (left) or Ki67 (right). Figure S11: Quantitative analysis of Ki67 positivity. Figure S12: Less than 5% Ki67 positive cells in non-proliferating regions of the untreated HCT116 xenograft.

## References

[1] Dagenais G.R., Leong D.P., Rangarajan S., Lanas F., Lopez-Jaramillo P., Gupta R., Diaz R., Avezum A., Oliveira G.B.F., Wielgosz A. (2020). Variations in common diseases, hospital admissions, and deaths in middle-aged adults in 21 countries from five continents (PURE): A prospective cohort study. Lancet.

[2] Abbema D.V., Vissers P., Vos-Geelen J., Lemmens V., Janssen-Heijnen M., Tjan-Heijnen V. (2019). Trends in Overall Survival and Treatment Patterns in Two Large Population-Based Cohorts of Patients with Breast and Colorectal Cancer. Cancers.

[3] Wong C.H., Siah K.W., Lo A.W. (2019). Estimation of clinical trial success rates and related parameters. Biostatistics.

[4] McIntyre R.E., Buczacki S.J., Arends M.J., Adams D.J. (2015). Mouse models of colorectal cancer as preclinical models. BioEssays News Rev. Mol. Cell. Dev. Biol..

[5] Gengenbacher N., Singhal M., Augustin H.G. (2017). Preclinical mouse solid tumour models: Status quo, challenges and perspectives. Nat. Rev. Cancer.

[6] Hutchinson L., Kirk R. (2011). High drug attrition rates—Where are we going wrong?. Nat. Rev. Clin. Oncol..

[7] Hung P.J., Lee P.J., Sabounchi P., Lin R., Lee L.P. (2005). Continuous perfusion microfluidic cell culture array for high-throughput cell-based assays. Biotechnol. Bioeng..

[8] Jeong S.Y., Lee J.H., Shin Y., Chung S., Kuh H.J. (2016). Co-Culture of Tumor Spheroids and Fibroblasts in a Collagen Matrix-Incorporated Microfluidic Chip Mimics Reciprocal Activation in Solid Tumor Microenvironment. PLoS ONE.

[9] Ayuso J.M., Virumbrales-Munoz M., Lacueva A., Lanuza P.M., Checa-Chavarria E., Botella P., Fernandez E., Doblare M., Allison S.J., Phillips R.M. (2016). Development and characterization of a microfluidic model of the tumour microenvironment. Sci. Rep..

[10] Hassell B.A., Goyal G., Lee E., Sontheimer-Phelps A., Levy O., Chen C.S., Ingber D.E. (2017). Human Organ Chip Models Recapitulate Orthotopic Lung Cancer Growth, Therapeutic Responses, and Tumor Dormancy In Vitro. Cell Rep..

[11] Nashimoto Y., Okada R., Hanada S., Arima Y., Nishiyama K., Miura T., Yokokawa R. (2020). Vascularized cancer on a chip: The effect of perfusion on growth and drug delivery of tumor spheroid. Biomaterials.

[12] Sontheimer-Phelps A., Hassell B.A., Ingber D.E. (2019). Modelling cancer in microfluidic human organs-on-chips. Nat. Rev. Cancer.

[13] Lohasz C., Frey O., Bonanini F., Renggli K., Hierlemann A. (2019). Tubing-Free Microfluidic Microtissue Culture System Featuring Gradual, in vivo-Like Substance Exposure Profiles. Front. Bioeng. Biotechnol..

[14] Komen J., Westerbeek E.Y., Kolkman R.W., Roesthuis J., Lievens C., van den Berg A., van der Meer A.D. (2020). Controlled pharmacokinetic anti-cancer drug concentration profiles lead to growth inhibition of colorectal cancer cells in a microfluidic device. Lab A Chip.

[15] Komen J., van Neerven S.M., van den Berg A., Vermeulen L., van der Meer A.D. (2021). Mimicking and surpassing the xenograft model with cancer-on-chip technology. EBioMedicine.

[16] Ivanova E., Kuraguchi M., Xu M., Portell A.J., Taus L., Diala I., Lalani A.S., Choi J., Chambers E.S., Li S. (2020). Use of Ex Vivo Patient-Derived Tumor Organotypic Spheroids to Identify Combination Therapies for HER2 Mutant Non-Small Cell Lung Cancer. Clin. Cancer Res. Off. J. Am. Assoc. Cancer Res..

[17] Petreus T., Cadogan E., Hughes G., Smith A., Reddy V.P., Lau A., O’Connor M.J., Critchlow S., Ashford M., O’Connor L.O. (2021). Tumour-on-chip microfluidic platform for assessment of drug pharmacokinetics and treatment response. Commun. Biol..

[18] Hachey S.J., Movsesyan S., Nguyen Q.H., Burton-Sojo G., Tankazyan A., Wu J., Hoang T., Zhao D., Wang S., Hatch M.M. (2021). An in vitro vascularized micro-tumor model of human colorectal cancer recapitulates in vivo responses to standard-of-care therapy. Lab A Chip.

[19] Gioeli D., Snow C.J., Simmers M.B., Hoang S.A., Figler R.A., Allende J.A., Roller D.G., Parsons J.T., Wulfkuhle J.D., Petricoin E.F. (2019). Development of a multicellular pancreatic tumor microenvironment system using patient-derived tumor cells. Lab A Chip.

[20] Nürnberg E., Vitacolonna M., Klicks J., von Molitor E., Cesetti T., Keller F., Bruch R., Ertongur-Fauth T., Riedel K., Scholz P. (2020). Routine Optical Clearing of 3D-Cell Cultures: Simplicity Forward. Front. Mol. Biosci..

[21] Liu W., Liu D., Hu R., Huang Z., Sun M., Han K. (2020). An integrated microfluidic 3D tumor system for parallel and high-throughput chemotherapy evaluation. Analyst.

[22] Hecker M., Ting M.S.H., Malmström J. (2018). Simple Coatings to Render Polystyrene Protein Resistant. Coatings.

[23] Liu V.A., Jastromb W.E., Bhatia S.N. (2002). Engineering protein and cell adhesivity using PEO-terminated triblock polymers. J. Biomed. Mater. Res..

[24] Wang J.C., Liu W., Tu Q., Ma C., Zhao L., Wang Y., Ouyang J., Pang L., Wang J. (2015). High throughput and multiplex localization of proteins and cells for in situ micropatterning using pneumatic microfluidics. Analyst.

[25] Ivanov D.P., Parker T.L., Walker D.A., Alexander C., Ashford M.B., Gellert P.R., Garnett M.C. (2014). Multiplexing spheroid volume, resazurin and acid phosphatase viability assays for high-throughput screening of tumour spheroids and stem cell neurospheres. PLoS ONE.

[26] Zhang R., Song X.Q., Liu R.P., Ma Z.Y., Xu J.Y. (2019). Fuplatin: An Efficient and Low-Toxic Dual-Prodrug. J. Med. Chem..

[27] Bankhead P., Loughrey M.B., Fernández J.A., Dombrowski Y., McArt D.G., Dunne P.D., McQuaid S., Gray R.T., Murray L.J., Coleman H.G. (2017). QuPath: Open source software for digital pathology image analysis. Sci. Rep..

[28] Robertson S., Acs B., Lippert M., Hartman J. (2020). Prognostic potential of automated Ki67 evaluation in breast cancer: Different hot spot definitions versus true global score. Breast Cancer Res. Treat..

[29] Nagaraju G.P., Alese O.B., Landry J., Diaz R., El-Rayes B.F. (2014). HSP90 inhibition downregulates thymidylate synthase and sensitizes colorectal cancer cell lines to the effect of 5FU-based chemotherapy. Oncotarget.

[30] Threatt S.D., Synold T.W., Wu J., Barton J.K. (2020). In vivo anticancer activity of a rhodium metalloinsertor in the HCT116 xenograft tumor model. Proc. Natl. Acad. Sci. USA.

[31] Liang J., Cheng Q., Huang J., Ma M., Zhang D., Lei X., Xiao Z., Zhang D., Shi C., Luo L. (2019). Monitoring tumour microenvironment changes during anti-angiogenesis therapy using functional MRI. Angiogenesis.

[32] de Bruijn M.T., Raats D.A., Hoogwater F.J., van Houdt W.J., Cameron K., Medema J.P., Borel Rinkes I.H., Kranenburg O. (2010). Oncogenic KRAS sensitises colorectal tumour cells to chemotherapy by p53-dependent induction of Noxa. Br. J. Cancer.

[33] Xu K., Chen G., Qiu Y., Yuan Z., Li H., Yuan X., Sun J., Xu J., Liang X., Yin P. (2017). miR-503-5p confers drug resistance by targeting PUMA in colorectal carcinoma. Oncotarget.

[34] Shelton J.W., Waxweiler T.V., Landry J., Gao H., Xu Y., Wang L., El-Rayes B., Shu H.K. (2013). In vitro and in vivo enhancement of chemoradiation using the oral PARP inhibitor ABT-888 in colorectal cancer cells. Int. J. Radiat. Oncol. Biol. Phys..

[35] Linnekamp J.F., Hooff S.R.V., Prasetyanti P.R., Kandimalla R., Buikhuisen J.Y., Fessler E., Ramesh P., Lee K., Bochove G.G.W., de Jong J.H. (2018). Consensus molecular subtypes of colorectal cancer are recapitulated in in vitro and in vivo models. Cell Death Differ..

[36] Li S., Chen Y., Zhang S., More S.S., Huang X., Giacomini K.M. (2011). Role of organic cation transporter 1, OCT1 in the pharmacokinetics and toxicity of cis-diammine(pyridine)chloroplatinum(II) and oxaliplatin in mice. Pharm. Res..

[37] Boughattas N.A., Hecquet B., Fournier C., Bruguerolle B., Trabelsi H., Bouzouita K., Omrane B., Lévi F. (1994). Comparative pharmacokinetics of oxaliplatin (L-OHP) and carboplatin (CBDCA) in mice with reference to circadian dosing time. Biopharm. Drug Dispos..

[38] Casalini T., Salvalaglio M., Perale G., Masi M., Cavallotti C. (2011). Diffusion and aggregation of sodium fluorescein in aqueous solutions. J. Phys. Chemistry. B.

[39] Modok S., Scott R., Alderden R.A., Hall M.D., Mellor H.R., Bohic S., Roose T., Hambley T.W., Callaghan R. (2007). Transport kinetics of four- and six-coordinate platinum compounds in the multicell layer tumour model. Br. J. Cancer.

[40] Paguirigan A.L., Beebe D.J. (2009). From the cellular perspective: Exploring differences in the cellular baseline in macroscale and microfluidic cultures. Integr. Biol. Quant. Biosci. Nano Macro.

[41] Hector A., Von Felten S., Schmid B. (2010). Analysis of variance with unbalanced data: An update for ecology & evolution. J. Anim. Ecol..

[42] NCI-60 Screening Methodology. https://dtp.cancer.gov/discovery_development/nci-60/methodology.htm.

[43] Heuberger J., Schmidt S., Derendorf H. (2013). When is protein binding important?. J. Pharm. Sci..

[44] Sun W., Li J., Zhou L., Han J., Liu R., Zhang H., Ning T., Gao Z., Liu B., Chen X. (2020). The c-Myc/miR-27b-3p/ATG10 regulatory axis regulates chemoresistance in colorectal cancer. Theranostics.

[45] Huxham L.A., Kyle A.H., Baker J.H., Nykilchuk L.K., Minchinton A.I. (2004). Microregional effects of gemcitabine in HCT-116 xenografts. Cancer Res..

[46] Bokemeyer C., Bondarenko I., Makhson A., Hartmann J.T., Aparicio J., Braud F.d., Donea S., Ludwig H., Schuch G., Stroh C. (2009). Fluorouracil, Leucovorin, and Oxaliplatin with and Without Cetuximab in the First-Line Treatment of Metastatic Colorectal Cancer. J. Clin. Oncol..

[47] Koopman M., Antonini N.F., Douma J., Wals J., Honkoop A.H., Erdkamp F.L., de Jong R.S., Rodenburg C.J., Vreugdenhil G., Loosveld O.J. (2007). Sequential versus combination chemotherapy with capecitabine, irinotecan, and oxaliplatin in advanced colorectal cancer (CAIRO): A phase III randomised controlled trial. Lancet.

[48] Forster J.C., Harriss-Phillips W.M., Douglass M.J., Bezak E. (2017). A review of the development of tumor vasculature and its effects on the tumor microenvironment. Hypoxia (Auckl. N.Z.).

[49] Kyle A.H., Baker J.H., Gandolfo M.J., Reinsberg S.A., Minchinton A.I. (2014). Tissue penetration and activity of camptothecins in solid tumor xenografts. Mol. Cancer Ther..

[50] Lenos K.J., Miedema D.M., Lodestijn S.C., Nijman L.E., van den Bosch T., Romero Ros X., Lourenco F.C., Lecca M.C., van der Heijden M., van Neerven S.M. (2018). Stem cell functionality is microenvironmentally defined during tumour expansion and therapy response in colon cancer. Nat. Cell Biol..

[51] Elmore S.A., Dixon D., Hailey J.R., Harada T., Herbert R.A., Maronpot R.R., Nolte T., Rehg J.E., Rittinghausen S., Rosol T.J. (2016). Recommendations from the INHAND Apoptosis/Necrosis Working Group. Toxicol. Pathol..

[52] Jusko W.J. (1973). A pharmacodynamic model for cell-cycle-specific chemotherapeutic agents. J. Pharmacokinet. Biopharm..

[53] Lobo E.D., Balthasar J.P. (2002). Pharmacodynamic modeling of chemotherapeutic effects: Application of a transit compartment model to characterize methotrexate effects in vitro. AAPS PharmSci.

[54] van der Heijden M., Miedema D.M., Waclaw B., Veenstra V.L., Lecca M.C., Nijman L.E., van Dijk E., van Neerven S.M., Lodestijn S.C., Lenos K.J. (2019). Spatiotemporal regulation of clonogenicity in colorectal cancer xenografts. Proc. Natl. Acad. Sci. USA.

[55] Stylianopoulos T., Martin J.D., Chauhan V.P., Jain S.R., Diop-Frimpong B., Bardeesy N., Smith B.L., Ferrone C.R., Hornicek F.J., Boucher Y. (2012). Causes, consequences, and remedies for growth-induced solid stress in murine and human tumors. Proc. Natl. Acad. Sci. USA.

[56] Ayers G.D., McKinley E.T., Zhao P., Fritz J.M., Metry R.E., Deal B.C., Adlerz K.M., Coffey R.J., Manning H.C. (2010). Volume of preclinical xenograft tumors is more accurately assessed by ultrasound imaging than manual caliper measurements. J. Ultrasound Med. Off. J. Am. Inst. Ultrasound Med..

