# Supplementary material for: The Effect of Dynamic, In Vivo-like Oxaliplatin on HCT116 Spheroids in a Cancer-on-Chip Model Is Representative of the Response in Xenografts
Source: Micromachines (Basel). 2022 May 6;13(5):739. doi: 10.3390/mi13050739 (PMC9146796; doi:10.3390/mi13050739)
Supplement: Supplementary file 1 [file micromachines-13-00739-s001.zip › micromachines-1687007 supplyment xml.pdf]

micromachines

Supplementary material to article

# The effect of dynamic, *in vivo*-like, oxaliplatin on HCT116 spheroids in a cancer-on-chip model is representative of the response in xenografts

**Job Komen[1], Sanne M. van Neerven[2], Elsbeth Bossink[2], Nina E. de Groot[2], Lisanne E. Nijman[2], Albert van den Berg[1], Louis Vermeulen[2] and Andries D. van der Meer[3].**

[1] BIOS Lab on a Chip group, MESA+ Institute for Nanotechnology, University of Twente, P. O. Box 217, 7500 AE Enschede, The Netherlands. E-mail: j.komen@utwente.nl
[2] Laboratory for Experimental Oncology and Radiobiology, Center for Experimental and Molecular Medicine, Cancer Center Amsterdam and Amsterdam Gastroenterology and Metabolism, Amsterdam University Medical Centers, 1105 AZ, Amsterdam, The Netherlands.
[3] Applied Stem Cell Technologies, TechMed Centre, University of Twente, P. O. Box 217, 7500 AE Enschede, The Netherlands.
* Correspondence: j.komen@utwente.nl

## Supplementary material

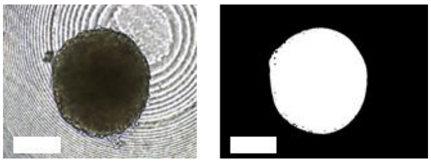

**Supplementary Figure S1. Spheroid size determination by the Fiji algorithm,** based on the method by Ivanov et al.[1]. The Fiji macro code used was as follows:

```
{
run("Enhance Contrast", "saturated=0.8")
run("16-bit");
setThreshold(75,255);
//setAutoThreshold("Yen");
setOption("BlackBackground", false);
run("Convert to Mask");
//getHistogram(0,hist,256);
//ratio = hist[255]/hist[0];
//run("Maximum...", "radius=3");
//run("Fill Holes");
//run("Minimum...", "radius=2");
run("Remove Outliers...", "radius=50 threshold=0 which=Bright");
run("Minimum...", "radius=2");
//run("Maximum...", "radius=30");
//run("Watershed");
}.
```

**Supplementary Table S1. Xenograft studies used for comparing growth inhibition and growth.** To compare oxaliplatin growth inhibition of HCT116 spheroids on-chip to xenografts, a literature search was conducted. Inclusion criteria were as follows: indexation in PubMed, analysis period and treatment start at a tumor starting volume of 50 mm³ or larger, subcutaneous placement of tumors, and more than one treatment cycle during the analysis period of 2–5 weeks.

| Author | Treatment | Start volume tumor (mm³) | 14 day volume (mm³) | Volume increase (vs. start) |
|---|---|---|---|---|
| De Bruijn et al. [2] | Control | 100 | 1060 | 10.5 |
| De Bruijn et al. [2] | Oxaliplatin | 100 | 450 | 4.5 |
| Liang et al. [3] | Control | 200 | 1000 | 5 |
| Liang et al. [3] | Oxaliplatin | 200 | 500 | 2.5 |
| Nagaraju et al. [4] | Control | 100 | 570 | 5.7 |
| Nagaraju et al. [4] | Oxaliplatin | 100 | 370 | 3.7 |
| Shelton et al. [5] | Control | 50 | 650 | 13 |
| Shelton et al. [5] | Oxaliplatin | 50 | 380 | 7 |
| Threatt et al. [6] | Control | 100 | 790 | 8 |
| Threatt et al. [6] | Oxaliplatin | 100 | 600 | 6 |
| Xu et al. [7] | Control | 100 | 570 | 6 |
| Xu et al. [7] | Oxaliplatin | 100 | 150 | 1.5 |
| Zhang et al. [8] | Control | 50 | 680 | 13 |
| Zhang et al. [8] | Oxaliplatin | 50 | 380 | 7 |

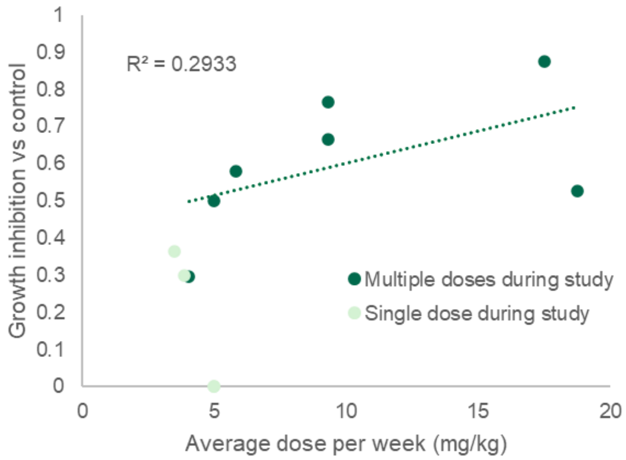


**Supplementary Figure S2. Growth inhibition from existing HCT116 xenograft studies plotted against dose per week.** Growth inhibition was between 30% and 80% for multiple doses. Dosages per week ranged from 4 to 20 mg/kg/week. There was a modest dose response relation for multiple dose studies, although the variability was relatively large and the explanatory power of dose was relatively low. Sources: multiple-dose studies [2–8]; single-dose studies [9–12].

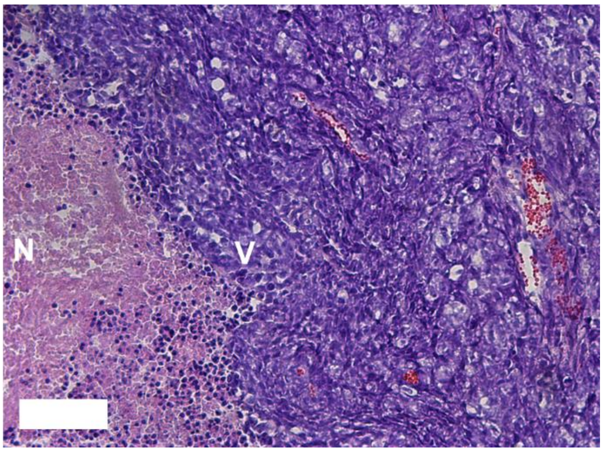


**Supplementary Figure S3. HCT116 xenograft tissue stained with hematoxylin–eosin (HE).** Viable HCT116 xenograft tissue (V) consisted of tumor tissue with limited differentiation, stroma and extracellular matrix, as there was mainly hematoxylin (blue) staining of nuclei and limited eosinophilic (pink) staining, implying limited extracellular matrix. Nonviable tissue (N) was characterized by nuclear shrinkage (pyknosis) and progressive loss of nuclei, which resulted in less hematoxylin (blue) staining and more pronounced eosinophilic staining (pink). Scale bar = 200 µm.

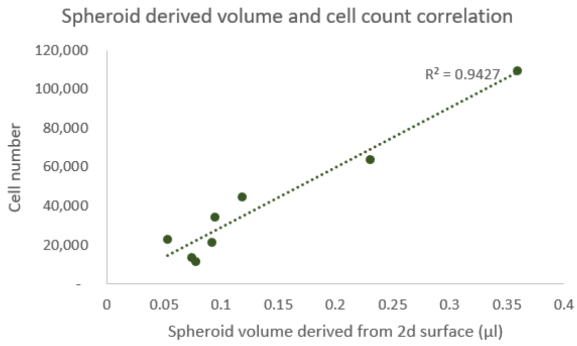


**Supplementary Figure S4. Correlation between image-based tumor volume derivation of the spheroids and automated cell count after trypsinization.**

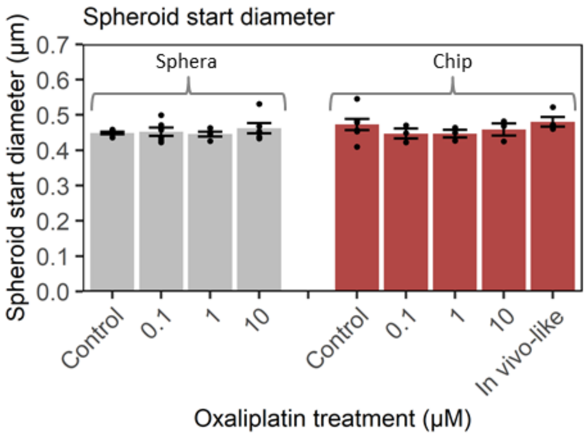


**Supplementary Figure S5. Equality of spheroid start diameter per treatment condition, on-chip and in the 96-well plate ("sphera").** Average start diameters per condition were between 0.45 mm and 0.48 mm.

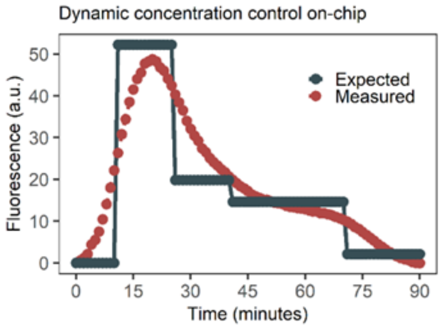


**Supplementary Figure S6. Validation of dynamic drug control on chip.** Dynamic solute concentration control was validated with fluorescein. Signal was evaluated with a Leica DM IRM fluorescence microscope. Flow was at 2 μL/min for all steps. Fluorescein concentrations at 10.5, 4.0, 2.9, and 0.4 μM were evaluated at 15, 15, 30, and 30 min, respectively. The last step was truncated. Fluorescein concentrations were chosen to match the concentrations of oxaliplatin of the first three steps of the flow schedule (Figure 2a) and the last step. The lag in concentration adjustments was due to Poisseuille flow [14].

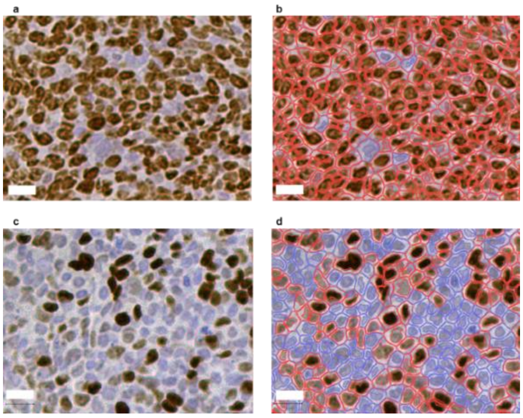

**Supplementary Figure S7. Ki67 (proliferation) quantification of HCT116 spheroid sections in Qupath.** Control spheroid (a,b) and spheroid treated with dynamic oxaliplatin after 2 days (c,d). Ki67-negative cells are encircled in blue, while Ki67-positive cells are encircled in red (b,d).

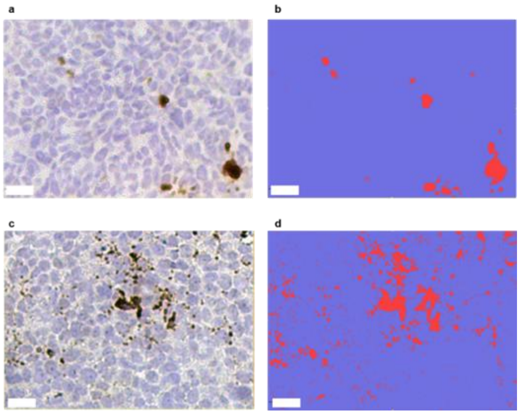

**Supplementary Figure S8. Detection of apoptosis marker cleaved caspase-3 (CC3).** Control spheroid (a,b) and spheroid treated with dynamic oxaliplatin after 2 days (c,d). A threshold for the DAB stain resulted in a CC3-positive area (c,d, in red). Scale bar = 20 um.

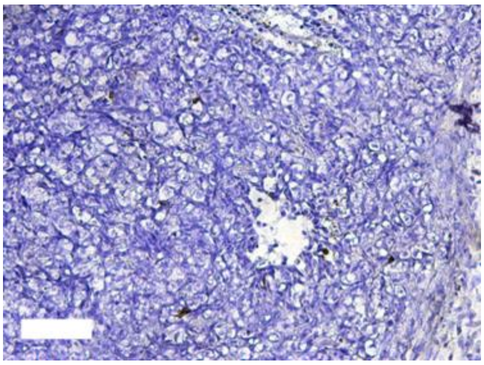

**Supplementary Figure S9. Limited cleaved caspase 3 (CC3) staining at the tumor edge of untreated xenografts.** Scale bar = 100 um.

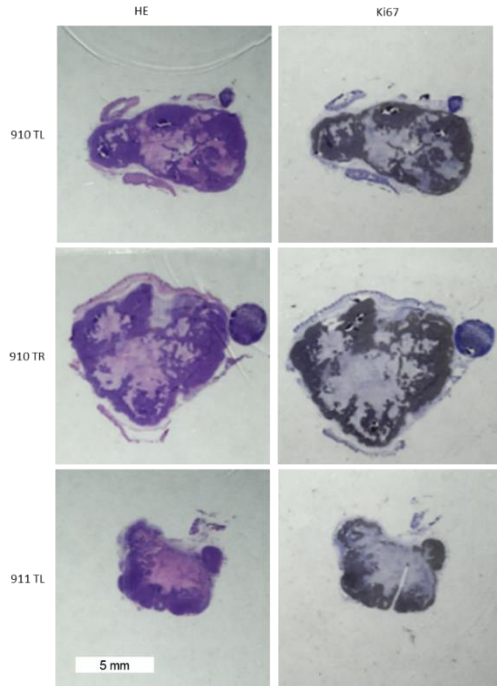


**Supplementary Figure S10. Macroscopic view of HCT116 xenografts stained with HE (left) or Ki67 (right).** Untreated HCT116 xenografts, at on average ~400 $mm^3$ (4.5 mm radius) volume. Hematoxylin (blue) staining was confined mostly to the outer parts of the spheroids, whereas central parts showed more eosin (pink) staining. Ki67 (DAB, brown) was also mostly confined to outer areas of the xenografts and correlated strongly to regions with intense hematoxylin staining.

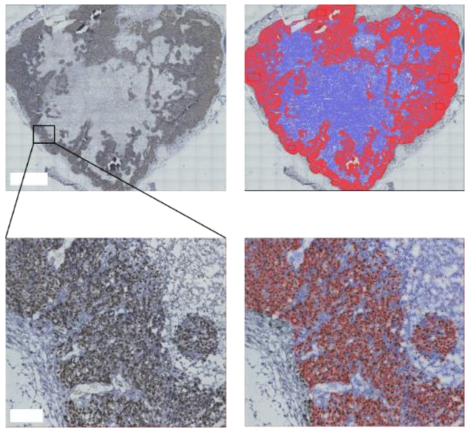

**Supplementary Figure S11. Quantitative analysis of Ki67 positivity.** Whole-slide analysis of xenograft tumor 910TR in Figure 10. Proliferating regions are shown in red, while nonproliferating regions are shown in blue. Scale bar = 2 mm top, 200 μm bottom.

*Supplementary Data to Supplementary Figures S9 and S10: Description of the Derivation of the Thickness of the Proliferating Shell of the Spheroid.*

To calculate the constant radius growth in the pharmacodynamic model, the thickness of the proliferating shell, consisting of Ki67-positive cells, needed to be estimated. First, in Qupath, cells were classified as Ki67-positive or -negative with nucleus detection parameters similar to Robertson et al [1].

As the proliferating cells were located more peripherally, the associated volume was not equal to the proportion of positive cells (40%) in the cross-section. An initial estimate would be to treat the Ki67-positive cells as perfectly located peripherally in a perfect sphere. Thus, 60% of Ki67-negative cells would occupy a fraction of 0.77 of the radius of a circle. If the nonproliferating radius had a fraction of 0.77 of the total radius, a sphere would have a non-proliferative volume of 46%. Hence, the proliferative volume would be 54%.

However, cells are not perfectly located peripherally. To more precisely estimate the associated volume, taking the location of cells into account, the associated relative volume per cell was estimated. An estimate was obtained by calculating the distance of cell from its $x$- or $y$-coordinate to the $x$ or $y$ centroid of the entire tumor. This distance provided a circumference around the axis of rotation. The circumference was representative of the relative volume, as the cell density for proliferating and non-proliferating regions was comparable at 8600 and 9500 per mm$^2$. The resulting proliferating volume estimate was then on average 46% (46%, 50%, and 43% for the three xenografts 910 TL, 910 TR, and 911 TL).

The volume of the xenografts was individually calculated by averaging the vertical and horizontal diameter, while calculating the volume as a perfect sphere. With the proportion of proliferative cells known, the (outer) radius of proliferating cells could be calculated. The average radius of the proliferating shell was calculated at 0.9 mm.

*Supplementary Data to Supplementary Figures S10 and S11: Derivation of Constant Radius Growth from On-Chip Growth and the Proliferating Shell of HCT116 Xenografts*

The average derived volume for the three xenograft control tumors was 432 mm$^3$, with a radius of 4.69 mm. With a proliferating outer shell of 0.9 mm, the proliferating volume was equal to 197 mm$^3$. Thus, 40% growth, based on spheroid growth in the first 2 days, before the formation of a necrotic core and decrease in the proliferation rate, of this

proliferating volume in 1 day resulted in a tumor volume of 505 $mm^3$. The radius was then 4.94mm. Therefore, the radius growth in 1 day was 0.25 mm/day.

When the spheroids were treated with in vivo-like oxaliplatin on-chip, growth stopped for 2 days, before subsequently recovering. The average growth rate over 1 week on-chip was 17%. Applying this growth rate to the proliferating shell thickness of 0.9 mm resulted in a constant radius growth of 0.11 mm/day (Supplementary Figures S9 and S10).

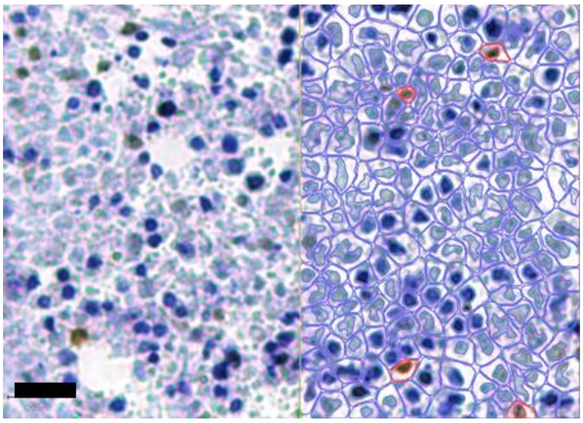

**Supplementary Figure S12. Image showing <5% Ki67-positive cells in nonproliferating regions.** Scale bar = 20 um.

## References


1. Ivanov, D.P.; Parker, T.L.; Walker, D.A.; Alexander, C.; Ashford, M.B.; Gellert, P.R.; Garnett, M.C. Multiplexing spheroid volume, resazurin and acid phosphatase viability assays for high-throughput screening of tumour spheroids and stem cell neurospheres. *PloS one* **2014**, *9*, e103817, doi:10.1371/journal.pone.0103817.
2. de Bruijn, M.T.; Raats, D.A.; Hoogwater, F.J.; van Houdt, W.J.; Cameron, K.; Medema, J.P.; Borel Rinkes, I.H.; Kranenburg, O. Oncogenic KRAS sensitises colorectal tumour cells to chemotherapy by p53-dependent induction of Noxa. *British journal of cancer* **2010**, *102*, 1254-1264, doi:10.1038/sj.bjc.6605633.
3. Liang, J.; Cheng, Q.; Huang, J.; Ma, M.; Zhang, D.; Lei, X.; Xiao, Z.; Zhang, D.; Shi, C.; Luo, L. Monitoring tumour microenvironment changes during anti-angiogenesis therapy using functional MRI. *Angiogenesis* **2019**, *22*, 457-470, doi:10.1007/s10456-019-09670-4.
4. Nagaraju, G.P.; Alese, O.B.; Landry, J.; Diaz, R.; El-Rayes, B.F. HSP90 inhibition downregulates thymidylate synthase and sensitizes colorectal cancer cell lines to the effect of 5FU-based chemotherapy. *Oncotarget* **2014**, *5*, 9980-9991, doi:10.18632/oncotarget.2484.
5. Shelton, J.W.; Waxweiler, T.V.; Landry, J.; Gao, H.; Xu, Y.; Wang, L.; El-Rayes, B.; Shu, H.K. In vitro and in vivo enhancement of chemoradiation using the oral PARP inhibitor ABT-888 in colorectal cancer cells. *International journal of radiation oncology, biology, physics* **2013**, *86*, 469-476, doi:10.1016/j.ijrobp.2013.02.015.
6. Threatt, S.D.; Synold, T.W.; Wu, J.; Barton, J.K. In vivo anticancer activity of a rhodium metalloinsertor in the HCT116 xenograft tumor model. *Proceedings of the National Academy of Sciences of the United States of America* **2020**, *117*, 17535-17542, doi:10.1073/pnas.2006569117.
7. Xu, K.; Chen, G.; Qiu, Y.; Yuan, Z.; Li, H.; Yuan, X.; Sun, J.; Xu, J.; Liang, X.; Yin, P. miR-503-5p confers drug resistance by targeting PUMA in colorectal carcinoma. *Oncotarget* **2017**, *8*, 21719-21732, doi:10.18632/oncotarget.15559.

8. Zhang, R.; Song, X.Q.; Liu, R.P.; Ma, Z.Y.; Xu, J.Y. Fuplatin: An Efficient and Low-Toxic Dual-Prodrug. *Journal of medicinal chemistry* **2019**, *62*, 4543-4554, doi:10.1021/acs.jmedchem.9b00128.
